# Longitudinal changes in nutritional parameters and resting energy expenditure in end-stage renal disease

**DOI:** 10.1590/2175-8239-JBN-2018-0169

**Published:** 2019-10-24

**Authors:** Mariana Cassani Oliveira, Marina Nogueira Berbel Bufarah, Daniela Ponce, André Balbi

**Affiliations:** 1 Universidade Estadual Paulista Júlio de Mesquita Filho, Faculdade de Medicina, Botucatu, SP, Brasil.

**Keywords:** Energy Metabolism, Calorimetry, Indirect, Renal Insufficiency, Chronic, Nutritional Status, Metabolismo Energético, Calorimetria Indireta, Insuficiência Renal Crônica, Estado Nutricional

## Abstract

**Aims:**

To evaluate the nutritional status, resting energy expenditure, caloric and protein intake, and evolution of biochemical parameters in three stages of chronic kidney disease: pre-dialytic, at the beginning of the dialysis treatment, and 30 days after starting treatment.

**Methods:**

The chi-square and Student’s t tests were used to compare the variables, and analysis of repeated measurements was used to compare the data obtained in the three moments evaluated. The results were discussed at the 5% level of significance.

**Results:**

We evaluated 35 patients, 60% female and 60% with diabetes mellitus. There was a decrease in midarm circumference and serum albumin. Inflammatory state and caloric and protein intake increased. There was no significant difference in resting energy expenditure in the three moments. The serum urea and serum albumin, handgrip strength, and protein consumption after 30 days from the start of dialysis were greater in the peritoneal dialysis patients, when compared to the hemodialysis population.

**Conclusion:**

there was a decrease in midarm circumference and serum albumin and an increase in protein intake after dialysis. The peritoneal dialysis patients had higher muscle strength, even with lower protein intake. Resting energy expenditure was not different between dialysis methods and the moments evaluated.

## Resumo

**Objetivos:**

Avaliar o estado nutricional, o gasto energético em repouso, o gasto calórico e proteico e a evolução dos parâmetros bioquímicos em três estágios da doença renal crônica: pré-dialítico, no início do tratamento dialítico e 30 dias após o início do tratamento.

**Métodos:**

O teste do qui-quadrado e o teste t de Student foram utilizados para comparar as variáveis, e a análise das medidas repetidas foi utilizada para comparar os dados obtidos nos três momentos avaliados. Os resultados foram discutidos ao nível de significância de 5%.

**Resultados:**

Foram avaliados 35 pacientes, 60% mulheres e 60% com diabetes mellitus. Houve uma diminuição na circunferência do terço médio do braço (CMB) e na albumina sérica. O estado inflamatório e a ingestão calórica e protéica aumentaram. Não houve diferença significativa no gasto energético em repouso nos três momentos. A ureia sérica e a albumina sérica, a força de preensão manual e o consumo de proteínas após 30 dias do início da diálise foram maiores nos pacientes em diálise peritoneal, quando comparados com a população em hemodiálise.

**Conclusão:**

houve diminuição da circunferência do terço médio do braço e na albumina sérica, e aumento da ingestão protéica após a diálise. Os pacientes em diálise peritoneal apresentaram maior força muscular, mesmo com menor consumo proteico. O gasto energético em repouso não foi diferente entre os métodos de diálise e os momentos avaliados.

## Introduction

In chronic kidney disease (CKD), nutritional status is affected by multiple factors and can trigger adverse consequences. It may be influenced by factors not only related to poor food intake but also those inherent to renal disease, such as anorexia, metabolic acidosis, anemia, hypervolemia, inflammation, poorly controlled diabetes, cardiovascular disease, and changes in energy expenditure^1^^-^ 3.

Indirect calorimetry (IC) is considered the gold standard method for determining resting energy expenditure (REE) because of its accuracy and high reproducibility^4^. Accuracy in the determination of REE from CKD individuals is important for the adequacy of nutritional needs, which has been the subject of debate among researchers in the area^3^.

Some studies using IC have evaluated whether uremia and dialysis could modify the REE of CKD patients, with conflicting results. The first studies showed that the REE of CKD non-dialytic and dialytic patients may be similar^5^^,^ 6 or significantly higher than that of healthy individuals^7^. More recent studies show that individuals with glomerular filtration rate (GFR) below 30mL/min present lower REE values^7^^,^ 8 and dialyzed patients present higher REE values when compared to the healthy population^3^^,^ 9.

End-stage CKD patients under catabolic conditions, such as poorly controlled diabetes mellitus^10^, severe hyperparathyroidism^11^, inflammation^12^, and metabolic syndrome^13^, present increased REE^3^^,^ 14. Individuals who underwent dialytic treatment, regardless of the method, had REE significantly higher than those on CKD conservative treatment^8^.

To date, no studies have evaluated the REE evolution of the same patients before and after the dialysis treatment initiation. In view of the above, the objectives of the present study were to evaluate the nutritional status and REE of CKD patients during the pre-dialysis phase, at the start of dialysis, and 30 days after.

## Methods

This was a prospective cohort study in which CKD patients were evaluated at the pre-dialysis outpatient clinic and started dialysis treatment at the same hospital.

Nutritional status and REE were assessed at three different moments of CKD: during pre-dialytic phase (phase 1 - P1), at dialysis initiation (phase 2 - P2: two to four days after the first dialysis section), and 30 days after the beginning of the dialytic therapy (hemodialysis or peritoneal dialysis) (phase 3 - P3).

### Patients

Patients with an estimated glomerular filtration rate (eGFR) ≤ 15 mL/min/1.73m^2^, older than 18 years, and who remained on dialysis for at least 30 days were included. Patients with neoplasms, those with reduced life expectancy evaluated by the nephrologist physician at the time of the dialysis appointment, and those who were unable to complete the REE assessment by IC were excluded.

### Laboratory data

Serum urea (mg/dL), urinary urea in an isolated sample of urine (mg/dL), serum creatinine (mg/dL), parathormone (pg/mL), total lymphocyte count (units/mm³), serum albumin (g/dL), C-reactive protein (mg/dL), and total cholesterol (mg/dL) were used.

The protein equivalent nitrogen appearance (PNA) was calculated with urinary urea from the 24-h urine test, collected for all the patients by the service laboratory.

eGFR was estimated by CKD-epi^9^ formula. According to the clinical symptomatology, dialysis therapy was indicated by the nephrologist.

The received dialysis dose was evaluated in hemodialysis and peritoneal dialysis patients at P3 by calculating the urea Kt/V^14^.

### Anthropometry and body composition

Subjects were weighed without shoes and with light clothes. Body mass index (BMI) was calculated as body weight divided by squared height. Midarm circumference (MAC) (cm), triceps skinfold thickness (TSF) (mm), and midarm muscle circumference (MAMC) (cm) were assessed by the Lohman method^15^.

Body cell mass (kg), extracellular mass (kg), intracellular water (L), extracellular water (L), total body water (L), and hydration state (L) were assessed by bioelectrical impedance analysis using a single-frequency tetrapolar technique (Biodynamics, 450). The electrodes were placed in the standard positions (two electrodes placed on the hand and wrist and another two positioned on the foot and ankle) in the opposite side of the vascular access, with the subject in supine position. Hydration status was calculated according to Watson^12^.

Handgrip strength (HGS) was evaluated by the Jamar^®^ hydraulic dynamometer. Subjects were instructed to sit with adducted shoulder, elbow flexed at 90º, and forearm neutral. The highest value of three readings was considered, with a rest period of 30 seconds between the tests with the same arm.

The anthropometry and bioimpedance measurements were made in the dominant arm or in the arm without the fistula. Patients on HD were submitted to measurements during the inter-dialytic day, and patients on PD were evaluated with empty cavity. These measurements were made by the same person in the three phases.

### Measurement of resting energy expenditure by indirect calorimetry

The REE evaluation was performed with IC, using a Cosmed Quark RMR. The instrument was calibrated before each measurement, according to the manufacturer’s instructions.

Patients were instructed to maintain regular medication, not to practice physical activity within 24 hours, and sleep for eight hours prior to testing. They were evaluated in the morning after 12 hours of overnight fasting. Patients under hemodialysis were submitted to the test during a day without hemodialysis; patients under peritoneal dialysis were submitted to the test during a morning after the dialysis session.

After 30 minutes of rest in reclining position, the subjects breathed for 20 minutes in the *canopy*, in silence, in a room with a temperature of 24°C. They were instructed to avoid hyperventilation, sudden movements, or falling asleep during the test.

For the measurement of oxygen consumption and carbon dioxide production, the first five minutes of the test were disregarded, and the final 15-minute average was considered. The REE was calculated according to the Weir equation^13^ without urinary urea nitrogen. Respiratory quotient (R) was calculated by the ratio between the volume of expired carbon dioxide and the oxygen consumed^13^.

### Caloric and protein intake

Food records of 72 hours were completed by subjects, during the three evaluation phases. Unfortunately, food records from the third evaluation stage were not obtained in sufficient quantity for statistical analysis.

As a substitute for protein intake, the PNA and normalized PNA (nPNA) were calculated from the current weight^14^.

### Statistical analysis

Variables comparison was performed by groups: pre-dialysis x start of dialysis (2 to 4 days after the first dialysis session) x late dialysis (30 days after the first dialysis session), and hemodialysis x peritoneal dialysis.

The chi-square and Student’s t tests were used to compare the variables. A normality test was performed to verify the data distribution. For those that presented a symmetrical distribution, the mixed model was adjusted for repeated measures by Tukey’s test for multiple comparisons and for the variables that presented an asymmetric distribution, a generalized linear model with gamma distribution was fitted, followed by multiple comparison test. *p* < 0.05 was considered significant. SAS program, version 9.2 was used.

## Results

**Figure 1:**
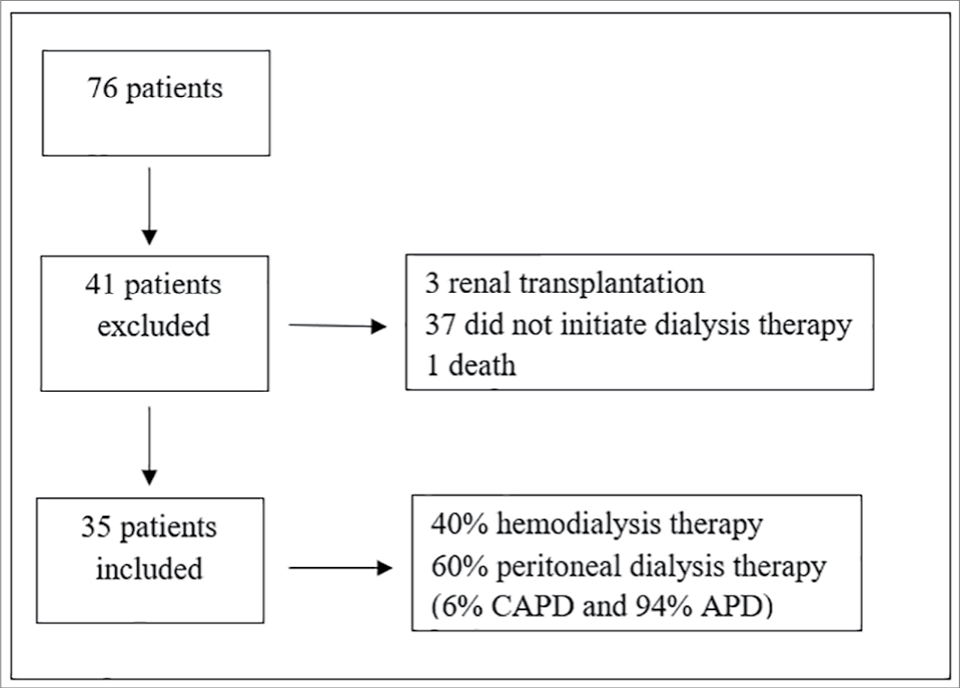
Patients inclusion and exclusion flowchart.

Seventy-six patients were evaluated. Thirty-five who had the dialytic therapy indication during outpatient follow-up patients were enrolled in the study (Figure 1).

The mean age of the participants was 61.2 ± 10.9 years, 60% female, 17% afro-descendants, and 60% with diabetes mellitus (DM). Thirty-four percent of these patients had four or more comorbidities, the most common was hypertension (37.4%) and DM (31.1%).

The first evaluation was performed, on average, 75 days before the beginning of dialysis therapy. The mean length of stay in the pre-dialysis outpatient clinic was 23.9 ± 15.9 months. Fourteen patients started renal replacement therapy by HD, being 65% female and with a mean age of 64.9 ± 7.5 years. Twenty-one patients started the PD, 57% female, with an average age of 58.7 ± 13.4 years. A significant difference was found between the age of the two groups (*p* = 0.04).

Table 1 shows the laboratory data at each moment evaluated. Serum albumin values were higher in the pre-dialytic phase and CRP values were significantly higher after dialysis initiation. Aside for the higher serum urea (P3: *p* = 0.007) and albumin (P3: *p* = 0.015) in PD, no other differences in laboratories parameters were found between dialysis modalities (data not shown).

**Table 1:** Comparison of laboratory variables in three time-points: pre-dialysis (P1), at start of dialysis (P2), and 30 days after dialysis (P3)

Laboratorial variables	P1 (n = 35)	P2 (n = 35)	P3 (n = 35)	*p*
eGFR (mL/min/1.73m^3^)	9.38 ± 0.7^a^^,^ b	5.8 ± 2.0	5.4 ± 2.7	0.00
Serum urea (mg/dL)	169.0 ± 58.9^a^	164.2 ± 56.6^c^	110.9 ± 48.5	< 0.00
Serum creatinine (mg/dL)	8.8 ± 2.5	8.4 ± 2.5	8.3 ± 3.0	0.70
Total cholesterol (mg/dL)	150.0 ± 33.3	159.8 ± 49.2	155.0 ± 77.9	0.74
PTH (mg/dL)	199.3 ± 117.0	282.1 ± 174.7	239.9 ± 147.0	0.09
Total lymphocyte count (%)	22.2 ± 5.6	19.1 ± 5.8	21.9 ± 7.7	0.09
Albumin (g/dL)	4.4 ± 0.9^a^^,^ b	3.4 ± 0.6	3.1 ± 0.7	< 0.00
CPR (mg/dL)	1.3 ± 1.1^a^^,^ b	2.9 ± 6.2	4.1 ± 8.1	0.04

eGFR: estimated glomerular filtration rate. PTH: parathyroid hormone. CPR: C-reactive protein

Statistical analysis:

a = P1 different from P3

b = P1 different from P2

c = P2 different from P3

Dialysis dose, measured by Kt/V, was collected only in the third moment. The mean Kt/V per session was 1.24 ± 0.34 for patients undergoing hemodialysis and weekly Kt/V of 1.45 ± 0.32 for those undergoing peritoneal dialysis, doses considered adequate by the local physicians.

Table 2 shows nutritional data of the patients at each moment of the study. Serum albumin and MAC values decreased significantly from the start of dialysis therapy. Caloric intake per kg of body weight, as well as the nPNA, were significantly higher after dialysis initiation when compared to the non-dialytic moment.

**Table 2:** Comparison of nutritional and food intake variables at three time-points: pre-dialysis (P1), at start of dialysis (P2), and 30 days after dialysis (P3)

Anthropometric and intake variables	P1 (n = 35)	P2 (n = 35)	P3 (n = 35)	*p*
Body weight (Kg)	71.7 ± 17.0	69.8 ± 16.4	68.5 ± 15.6	0.72
BMI (Kg/m^2^)	28.1 ± 5.7	27.3 ± 5.5	26.8 ± 5.3	0.64
MAC (cm)	31.0 ± 4.9	29.6 ± 5.1	28.4 ± 5.1	0.11
TSF (mm)	19.1 ± 9.0	18.0 ± 8.9	16.5 ± 7.6	0.43
MAMC (cm)	30.4 ± 4.7^a^	29.0 ± 4.9^b^	25.4 ± 5.6	0.00
HGS (Kg)	17.2 ± 8.6	15.9 ± 7.6	16.5 ± 7.5	0.81
Phase angle (º)	6.1 ± 0.9	6.0 ± 1.2	6.1 ± 1.2	0.89
Body cell mass (kg)	22.5 ± 6.4	21.0 ± 5.4	20.8 ± 5.9	0.47
Extracellular mass (kg)	27.1 ± 6.9	26.1 ± 5.8	25.0 ± 5.3	0.38
Intracellular water (L)	19.3 ± 5.3	18.1 ± 4.8	17.8 ± 4.8	0.47
Extracellular water (L)	18.1 ± 4.9	16.8 ± 4.0	16.2 ± 3.7	0.21
Hydration state (L)	1.1 ± 4.6	0.4 ± 3.1	0.2 ± 2.7	0.57
PNA (g/day)	41.9 ± 16.3	35.5 ± 14.1	34.1 ± 17.4	0.26
nPNA (g/kg/day)	0.5 ± 0.2	0.5 ± 0.2^b^	0.6 ± 0.2	0.04
Calories (Kcal)	1214.0 ± 547.3	1294.7 ± 366.8	-	0.29
Calories (Kcal/kg)	17.4 ± 8.7	20.7 ± 6.9	-	0.00
Proteins (g)	50.4 ± 21.0	51.2 ± 21.4	-	0.81
Proteins (g/kg)	0.71 ± 0.31	0.8 ± 0.4	-	0.07

BMI: body mass index; MAC: midarm circumference; TSF: triciptal skinfold; MAMC: midarm muscle circumference; HGS: handgrip strength; PNA: protein equivalent of nitrogen appearance; nPNA: normalized PNA.

Statistical analysis:

a = A1 different from A3.

b = A2 different than A3.

PD patients had lower protein intake (P3: *p* = 0.010) evaluated by PNA, higher albumin levels (P3: *p* = 0.015), and higher muscular strength (P2: *p* = 0.036; P3: *p* = 0.034) compared with HD patients. No differences were found between PD and HD patients for the others nutritional parameters (data not shown).

**Figure 2:**
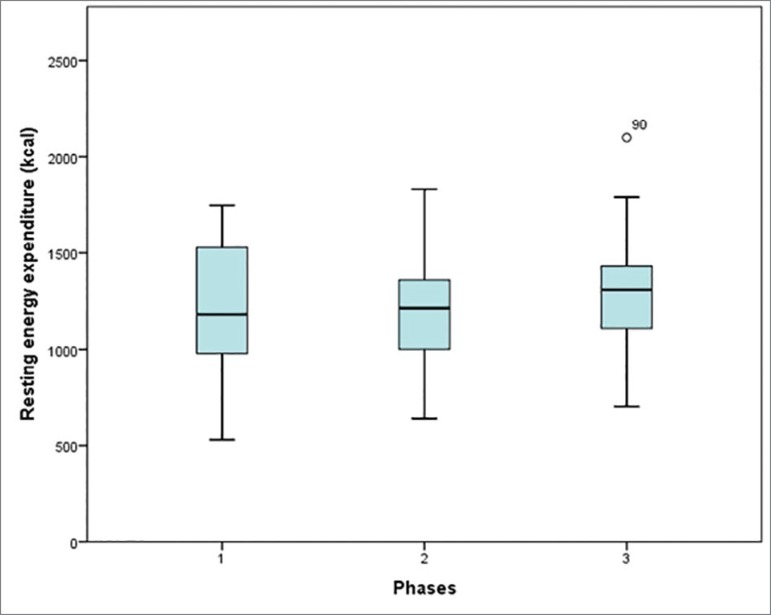
Comparisons of energy balance at three time-points: predialysis (M1), at start of dialysis (M2), and 30 days after initiation of dialysis (M3).

Figure 2 shows the comparisons of energy expenditure variables presented by the patients at each evaluated moment, as showed in tables in our last study^16^. No statistically significant differences were found in REE values among the three moments measured with the IC test. No difference was found in REE between HD and PD (*p* = 0.795) patients (data not shown).

## Discussion

In the present study, we evaluated CKD stage 5 patients on hemodialysis or peritoneal dialysis in the pre-dialytic stage, at dialysis start, and one month after the beginning of dialysis therapy. The REE values were not different between the groups analyzed.

The progression of CKD leads to the final stage of the disease, in which the GFR is less than or equal to 15mL/min/1.73m^2^. At this stage, the indication of renal replacement therapy is performed according to clinical symptoms such as azotemia, hyperkalemia, inappetence, weight loss, and protein-energy wasting^17^^,^ 18.

Reduced serum albumin is considered a strong predictor of morbidity and mortality in the CKD population in all stages and, with other clinical parameters, is an indicative of dialysis therapy initiation^19^^,^ 20. In addition, hypoalbuminemia may indicate inflammation and depletion of nutritional status^21^.

In the present study, the mean value of albumin was adequate in P1 for all patients, decreasing significantly from conservative care to start of dialysis. However, CRP values also increased significantly, which may mask alterations in nutritional status considering only serum albumin variation. The peritoneal albumin loss is an important factor of hypoalbuminemia in PD^22^.

It is known that albumin loss is greater in PD and, despite of this, the HD patients have a lower serum level of albumin. This can be explained by the better residual renal function of PD patients.

Reduction of MAC values, regardless of body weight and state of hydration and inflammation, may suggest decreased muscle mass. Reduction of lean mass, inflammatory state, and hypoalbuminemia are considered risk factors for cardiovascular disease, increased mortality, and rapid progression of CKD^20^.

MAC values in our study reduced progressively from P1 to P3 in all patients, and may be associated with insufficient protein intake, below that recommended for the conservative and dialytic treatment phases.

Even with protein intake estimated by the nPNA below the recommended intake for the pre-dialytic stage of CKD, from 0.6 to 0.8g/kg/day^9^, patients showed normal serum albumin levels in the first evaluation. Protein intake, evidenced by the nPNA, was reduced in the pre-dialytic phase and at dialysis indication but was significantly increased after the dialysis initiation, even though it was lower than that recommended by the NKF-DOQI nutritional guidelines (1.2 g protein/kg/day)^21^. Increasing protein intake in dialysis is a strong recommendation for recovery and maintenance of patients’ nutritional status in view of protein loss during dialysis sessions, regardless of the dialytic method^23^.

Caloric intake was also below the recommendation for end-stage CKD phase (30 to 35 kcal/kg/day^21^), with a mean of 17.4 ± 8.7 kcal/kg/day at P1 and 20.7 ± 6.9 kcal/kg/day at P2, suggesting that both inadequacies could be possible causes for muscle mass lost. It is clear in the literature that, in order to avoid nutritional depletion caused by a low-protein diet, adequate caloric supply is indispensable^23^^,^ 24.

Although the patients presented PNA increase at P3, the monitoring period was too short for clinical repercussion of increased protein intake. In addition, food records obtained at P3 were not enough for comparison and analysis of caloric and protein intake.

Despite the significant MAC reduction suggesting the loss of muscle mass, this loss was not reflected in REE. Several metabolic abnormalities are involved in REE alteration in patients with CKD, such as diabetes mellitus, inflammation, and hyperparathyroidism. Other components, such as the amount of muscle mass, serum albumin values, age, sex, and race may also alter energy expenditure^8^^,^ 25.

Avesani et al. showed REE was significantly higher in patients with pre-dialytic CKD and subclinical inflammation (CRP > 0.5 mg/dL) than in patients with CRP levels below 0.14 mg/dL, even when adjusted for sex, age, and lean mass. In the present study, the significant increase in CRP did not influence REE values.

Skouroliakou et al. showed that REE of hemodialysis patients is determined by the amount of lean mass: after adjusting for lean mass, the authors observed that REE of this population was significantly higher than the control group. The amount of lean mass was the only significant determinant of REE in that study, suggesting protein catabolic status and risk of malnutrition^25^.

The progression of CKD is characterized by worsening of uremic symptoms, leading to poor protein intake, and thyroid and inflammatory changes, which are associated with increase of nutritional risk. Nevertheless, no association was found between depletion of nutritional status and change in REE in our study.

These results show the difficulty of assessing the catabolic state, a risk for malnutrition in end-stage CKD patients. Adequate nutritional support is essential for maintenance of proper nutritional status and maintenance of muscle mass^9^^,^ 10.

Even in the presence of MAC reduction and protein intake below the recommended values, there was no significant repercussion in REE. The short follow-up time and the small number of patients might be study limitations. In addition, frequent nutritional monitoring by nutritionists at the outpatient clinic may have great importance in minimizing negative nutritional outcomes.

## Conclusions

In conclusion, this study showed a progressive decrease of the MAC and serum albumin, as well as an inadequate caloric and protein intake in the pre-dialytic period, which could lead to nutritional depletion, such as lean body mass loss and serum albumin reduction. There was no change in REE of these patients in the transitional period between pre-dialysis and dialysis, and between hemodialysis and peritoneal dialysis treatments.

New studies with longer follow-up periods after the initiation of dialysis therapy are necessary to identify possible nutritional status and energetic balance changes in the long term. In addition, evaluation of dietary interventions are needed to achieve adequate caloric and protein intake.
